# Physically active pregnancies: Insights from the placenta

**DOI:** 10.14814/phy2.16104

**Published:** 2024-06-14

**Authors:** Kristi B. Adamo, Alexandra D. Goudreau, Abbey E. Corson, Meaghan L. MacDonald, Nicholas O'Rourke, Velislava Tzaneva

**Affiliations:** ^1^ School of Human Kinetics, Faculty of Health Sciences University of Ottawa Ottawa Ontario Canada; ^2^ Department of Obstetrics and Gynecology, Faculty of Medicine University of Ottawa Ottawa Ontario Canada; ^3^ Department of Experimental Medicine, Faculty of Medicine and Health Sciences McGill University Montreal Quebec Canada

**Keywords:** lipids, mitochondria, nutrient transport, physical activity, placenta, pregnancy

## Abstract

Physical activity (PA) positively influences pregnancy, a critical period for health promotion, and affects placental structure and function in ways previously overlooked. Here, we summarize the current body of literature examining the association between PA, placenta biology, and physiology while also highlighting areas where gaps in knowledge exist. PA during pregnancy induces metabolic changes, influencing nutrient availability and transporter expression in the placenta. Hormones and cytokines secreted during PA contribute to health benefits, with intricate interactions in pro‐ and anti‐inflammatory markers. Extracellular vesicles and placental “‐omics” data suggest that gestational PA can shape placental biology, affecting gene expression, DNA methylation, metabolite profiles, and protein regulation. However, whether cytokines that respond to PA alter placental proteomic profiles during pregnancy remains to be elucidated. The limited research on placenta mitochondria of physically active gestational parents (gesP), has shown improvements in mitochondrial DNA and antioxidant capacity, but the relationship between PA, placental mitochondrial dynamics, and lipid metabolism remains unexplored. Additionally, PA influences the placenta‐immune microenvironment, angiogenesis, and may confer positive effects on neurodevelopment and mental health through placental changes, vascularization, and modulation of brain‐derived neurotrophic factor. Ongoing exploration is crucial for unraveling the multifaceted impact of PA on the intricate placental environment.

## 1 INTRODUCTION

The Developmental Origins of Health and Disease (DOHaD) framework posits that environmental perturbations before conception, in utero, and in the early postnatal period can alter offspring development and influence health and disease risk (Barker, 2004; Stiemsma & Michels, 2018). Even transient diseases or conditions of pregnancy, such as gestational diabetes (GDM), preeclampsia, and excessive gestational weight gain (GWG), can have long‐term implications for chronic disease susceptibility for gestational parents (gesP) and their offspring, unless otherwise prevented (Adamo et al., 2012). Pregnancy represents a unique period when a variety of factors coalesces to make disease prevention more attainable (Sattar, 2002). One extraordinarily successful disease prevention strategy is habitual engagement in physical activity (PA).

**FIGURE 1:**
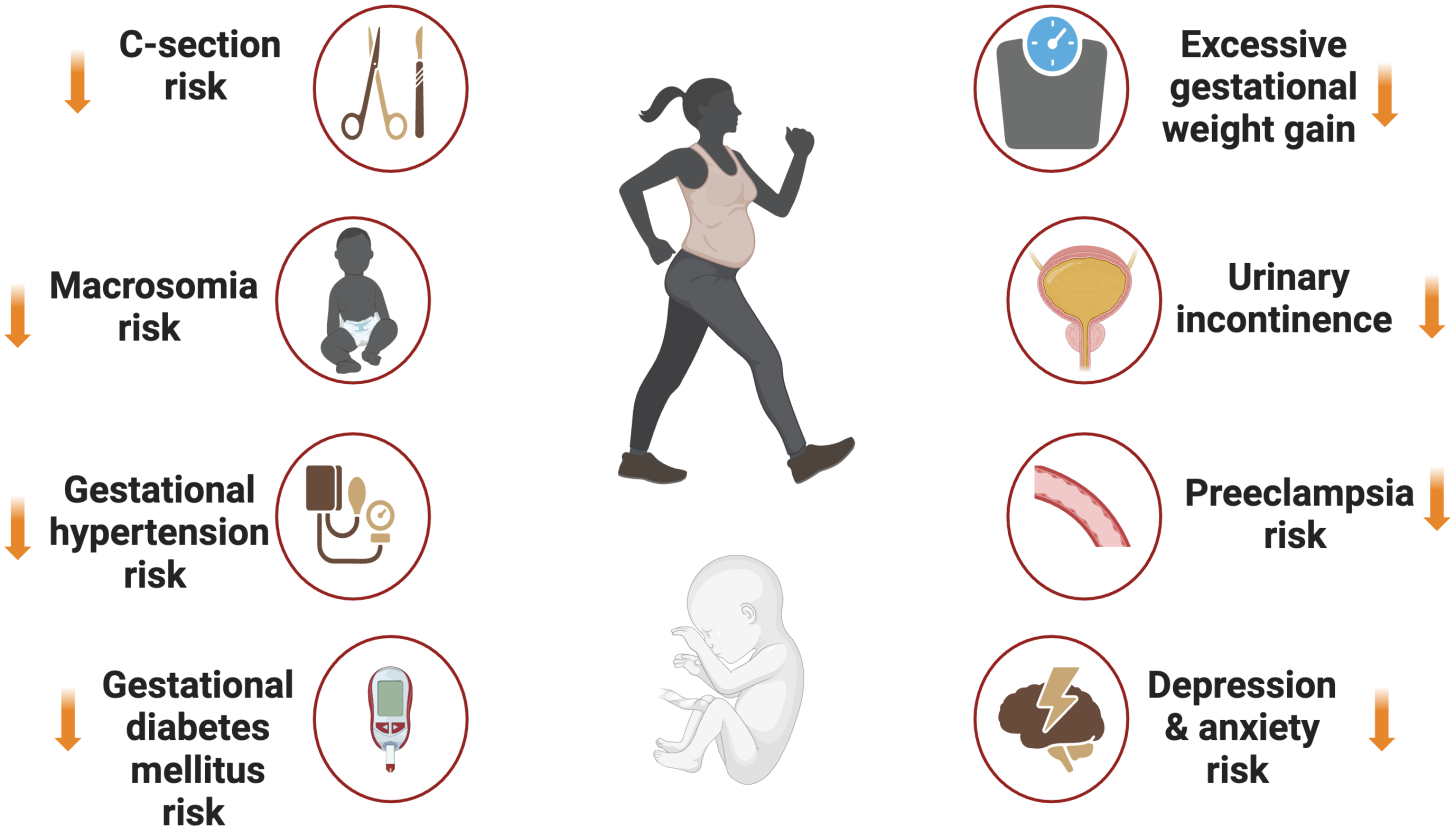
Gestational physical activity associations with pregnancy and birth outcomes.

Traditionally, obstetrical research is heavily biased toward investigations of *pathological exposures* (i.e., smoking/drug use, diabetes, and obesity) on placental‐fetal biology. In stark contrast, exposures that decrease disease susceptibility and promote health maintenance, such as exercise/PA, remain severely unstudied, despite their strong association with global disease risk reduction (Ekelund et al., 2015; Pedersen & Saltin, 2015). During the late 19th century, extending into the early 20th century, the common medical opinion was that pregnant women should avoid exertion and fatigue (Downs et al., 2012; Lotgering, 2014; Sprague, 2004; Wood, 2016). Most of the early published guidelines for pregnant individuals were unscientific and reinforced the notion that females were weak and frail (Bergman, 2002; Wood, 2016). Contrary to historical beliefs, PA is not the cause of miscarriage (Davenport, Kathol, et al., 2019), does not lead to premature births or fetal growth restriction (FGR) (Davenport, Meah, et al., 2018), and does not result in increases in core body temperature exceeding congenital anomaly threshold (Davenport, Yoo, et al., 2019). In fact, a set of rigorously developed, evidence‐based guidelines, supported by a set of 12 systematic reviews, discredited the idea that it is unsafe to be physically active during pregnancy (Mottola et al., 2018). Collectively, data illustrates that regular PA reduces the risk of many pregnancy complications, as shown in Figure 1.

Whereas exercise biologists have identified adaptive responses in virtually every human tissue/system (Heinonen et al., 2014), the placenta, the pivotal organ of pregnancy, has been largely ignored. Given that placenta structure and function predict pregnancy and neonatal health metrics (Albu et al., 2014; Bhattacharjee, Mohammad, & Adamo, 2021; Kingdom et al., 2000) and that PA independently improves these metrics, it is logical to investigate the placental response to PA. Once considered inconsequential afterbirth, the placenta is now considered the “black box” of pregnancy, housing important data about the gestational period. Its functions extend beyond mere nutrient exchange and waste elimination, involving complex processes of hormonal regulation, immune modulation, and metabolic adaptation. Functioning as a bridge between the parental and fetal circulations, the placenta adapts to meet the evolving needs of the growing fetus, making it a highly robust and multifunctional organ. While its critical role is inarguable, recent years have witnessed an increased interest in understanding the impact of gestational PA on placental structure and function. Since the seminal work by Clapp et al. (2002) on the positive effect of PA on placenta development and fetal growth (Jackson et al., 1995), our lab and others have unveiled a captivating dynamic between PA and the placenta. Here, we review these relationships and the known or potential implications for gesP health and fetal development.

## 2 NUTRIENT TRANSPORT

Glucose and other nutrients (including lactic acid, free amino acids, free fatty acids, and ketone bodies) are transported by the placenta to support fetal growth and development (Robertson & Karp, 1976). Nutrient transport into the placenta is determined by two factors: (i) availability of nutrients and (ii) the ability of the nutrients to be transported. The impact of pregnancy and PA on the gesP metabolism has previously been reviewed (Bessinger & Mcmurray, 2003). Briefly, plasma levels of glucose decrease while free fatty acids (FFA) and triglycerides (TG) increase after a bout of exercise, without changes in respiratory exchange ratio. A clear trend has not emerged for protein metabolism (Bessinger & Mcmurray, 2003). These modifications with PA suggest that nutrient availability changes, but utilization for energy production does not.

Emerging evidence suggests that the placenta can sense the availability of nutrients and adapt to meet the needs of the fetus and optimize its development (Jansson & Powell, 2013; Sandovici et al., 2012). Thus, the adjustment in nutrient availability due to PA may lead to shifts in nutrient transporter expression. In 2015, Brett et al. (2015) measured gene expression of nutrient transporters in the placenta from physically active individuals compared to physically inactive individuals. When moderate‐to‐vigorous PA (MVPA) guidelines were met in the second trimester, there was a 1.85‐fold lower fatty acid transporter FATP4 gene expression (Brett et al., 2015). Interestingly, placentas from physically active individuals have shown higher protein expression of this fatty acid transporter than physically inactive individuals (Hutchinson et al., 2020). Contrarily, GLUT1 gene and protein expression have displayed similar nonsignificant patterns in response to chronic PA (Brett et al., 2015; Hutchinson et al., 2020); however, the localization of this glucose transporter may be affected by gesP PA. GLUT1 proteins measured by immunohistology in placenta from habitually active individuals had a stronger presence in the fetal endothelium and the apical border of the syncytium (Hutchinson et al., 2020). In contrast, the inactive placenta showed a more even transporter distribution across the syncytium (Hutchinson et al., 2020). Lastly, amino acid transporter SNAT2 gene expression was 1.68‐fold higher (Brett et al., 2015) with no detected changes in SNAT transporter protein expression in the physically active group (Hutchinson et al., 2020).

**FIGURE 2:**
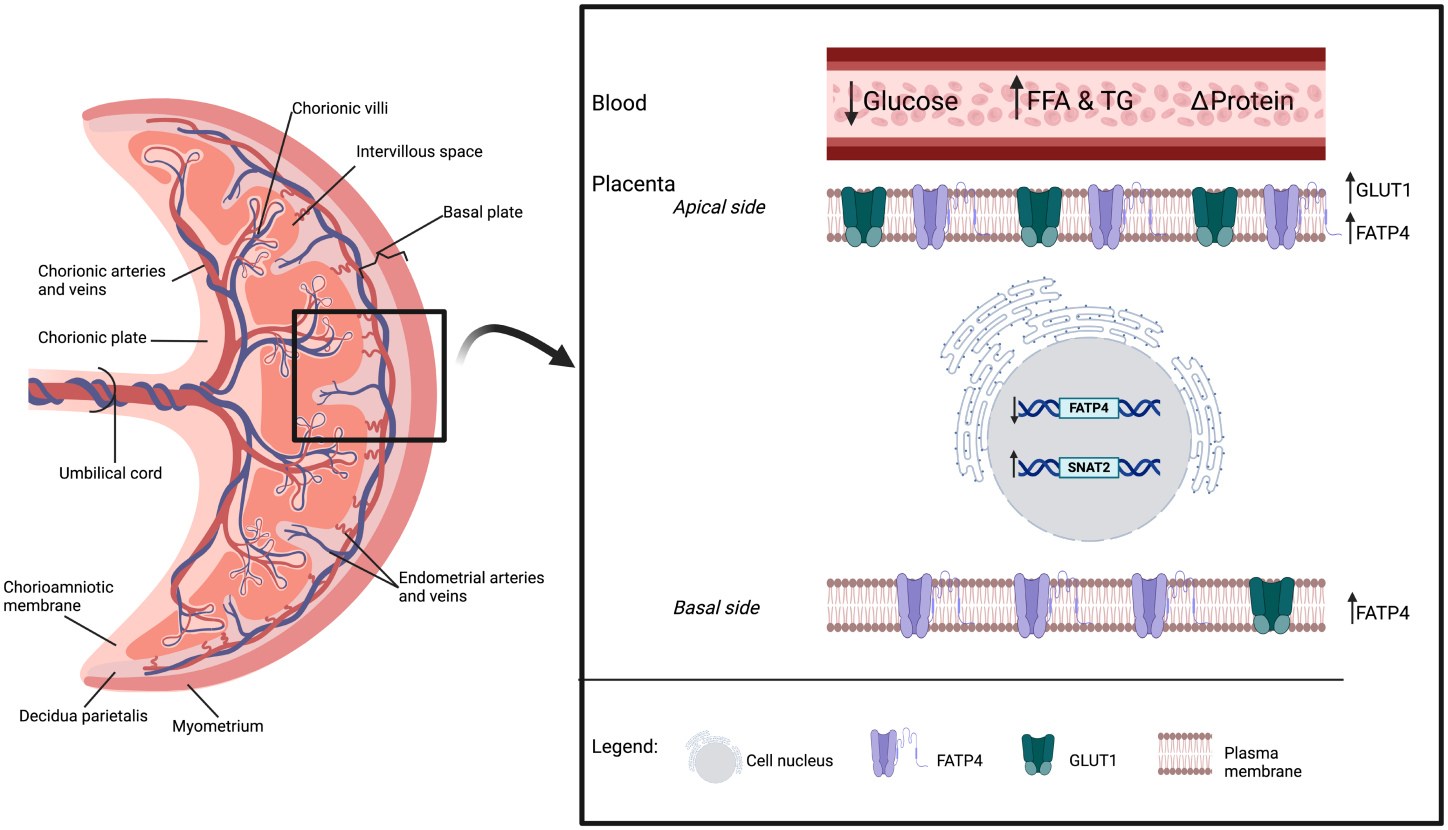
Differences in nutrient transport gene and protein expression in the placenta of physically active individuals.

Intriguingly, protein expression may not follow the same trend previously discussed when exposed to acute PA. An in‐vitro model using BeWO cells exposed to serum from the gesP, collected after 30 min of moderate‐intensity walking, showed increased GLUT1 protein but not SNAT1 or FATP4 (Hutchinson et al., 2020). In summary, changes in the availability of nutrients during prenatal PA may elicit gene and protein expression transformations to refine the delivery of essential building blocks to the fetus (Figure 2).

## 3 SECRETED FACTORS

A potential mechanism for the health benefits of gesP PA may be the endocrine, autocrine, or paracrine effects of secreted molecules. The hormonal responses to PA in pregnant individuals have previously been summarized (Bessinger, 2013; Bessinger & Mcmurray, 2003; Pahlavani et al., 2023). Other hormones, such as growth factors FGF21, EPO, and BDNF, have been shown to rise in pregnant individuals after a 30‐min moderate‐intensity treadmill walking compared to nonpregnant controls (Hutchinson et al., 2019). These hormones are thought to act in energy metabolism, erythropoiesis, and nervous system regulation, respectively (Sattar, 2002). Moreover, irisin, a hormone implicated in lipid homeostasis, substantially increased following a bout of PA during pregnancy (Szumilewicz et al., 2019). Over a 6‐week training intervention, irisin continued to be higher in training (vs. non‐training) individuals at the third and sixth week (Szumilewicz et al., 2019).

The intricate relationship between PA and cytokine levels during pregnancy reveals a nuanced interplay between pro‐ and anti‐inflammatory markers. Acosta‐Manzano et al. (2019) found that those who engaged in habitual PA through pregnancy had reduced levels of pro‐inflammatory IL‐1β and TNF‐a following an aerobic‐resistance training intervention; however, this association was attenuated when controlling for confounding factors (e.g. circulating cytokines at baseline, PA levels, and parental demographics) (Acosta‐Manzano et al., 2019). Interestingly, venous cord blood exhibited a consistent decrease in TNF‐α levels (Acosta‐Manzano et al., 2019). Unlike IL‐1β and TNF‐α, the pro‐inflammatory cytokine IL‐15 demonstrated a notable increase in pregnant individuals after 30 min of treadmill walking, with no such effect observed in nonpregnant controls (Hutchinson et al., 2019).

There are similarly mixed results in studies that examined anti‐inflammatory cytokines. An acute bout of walking induced an increase in fractalkine in both pregnant and nonpregnant participants, with levels remaining unchanged in parental serum profile in early, mid, and late pregnancy, as well as arterial and venous cord blood serum at delivery (Acosta‐Manzano et al., 2019; Hutchinson et al., 2019). Studies investigating the anti‐inflammatory cytokine IL‐10 are inconclusive. Individuals who engaged in more MVPA early in pregnancy demonstrated a higher IL‐10, while increased light‐to‐vigorous PA was associated with IL‐10 in the same period of time (Steckle et al., 2021; Van Poppel et al., 2014). Discrepancies continued with other studies not observing any notable differences (Acosta‐Manzano et al., 2019, 2020).

The pleiotropic cytokine IL‐6 displayed diverse responses to PA during pregnancy. A brief period of walking left IL‐6 levels unchanged (Hutchinson et al., 2019). Parental serum and venous cord blood serum levels showed no substantial changes in response to a training program, while arterial cord blood exhibited decreased IL‐6 levels (Acosta‐Manzano et al., 2019). Paradoxically, active individuals from a different study, identified by reduced sedentary time, displayed higher IL‐6 levels in midpregnancy (Nayak et al., 2016). Several studies found no sizeable activity‐driven differences in IL‐6 levels (Acosta‐Manzano et al., 2020; Hutchinson et al., 2019; Steckle et al., 2021). Conflicting findings may be related to differences in study design (e.g., prospective vs. retrospective), sample characteristics (e.g., BMI status), and independent variables (e.g., acute bouts of exercise versus habitual PA). Thus, there is a strong need for future research on different forms of PA in diverse populations.

As for the pro‐inflammatory cytokines, MCP‐1, IL‐8, and IFN‐γ studies have not found differences associated with PA (Acosta‐Manzano et al., 2019; Dekker Nitert et al., 2015). Evidence for the role of notable and well‐characterized cytokines in exercise sciences, such as IL‐4, IL‐12, IL‐17, IL‐23, and IL‐21, is lacking in the pregnant population. Further investigation is warranted to comprehensively understand the impact of PA on a broader spectrum of cytokine responses in this population.

Another secretory factor that may play a mechanistic role in the health benefits associated with gesP PA are extracellular vesicles (EVs). Most EV research has been conducted in nonpregnant populations; thus, current findings may not apply to our population of interest. Of the limited research examining EVs in the pregnant population, baseline levels of small EVs isolated from the plasma (10–120 nm) were higher in pregnant individuals than nonpregnant individuals (Mohammad, Hutchinson, et al., 2021). After a 30‐min bout of moderate‐intensity exercise, serum levels of small EVs were significantly higher in gesP than controls (Mohammad, Hutchinson, et al., 2021). Conversely, levels of circulating large EVs, specifically endothelial and platelet EVs, were not impacted by an acute bout of walking, nor associated with markers of cardiorespiratory fitness (Abolbaghaei et al., 2023).

In summary, gestational PA is implicated in regulating hormonal growth factors, inflammation, and EVs, feasibly modifying fundamental processes for both placental and fetal development, such as including energy metabolism, erythropoiesis, nervous system regulation, lipid homeostasis, and inflammatory responses. Future research is needed to discern the effects of pathway interplay and extraneous factors that might account for differential regulation of secreted factors, and their potential downstream effects on placental and fetal development.

## 4 PLACENTA ‐*OMICS*

**FIGURE 3:**
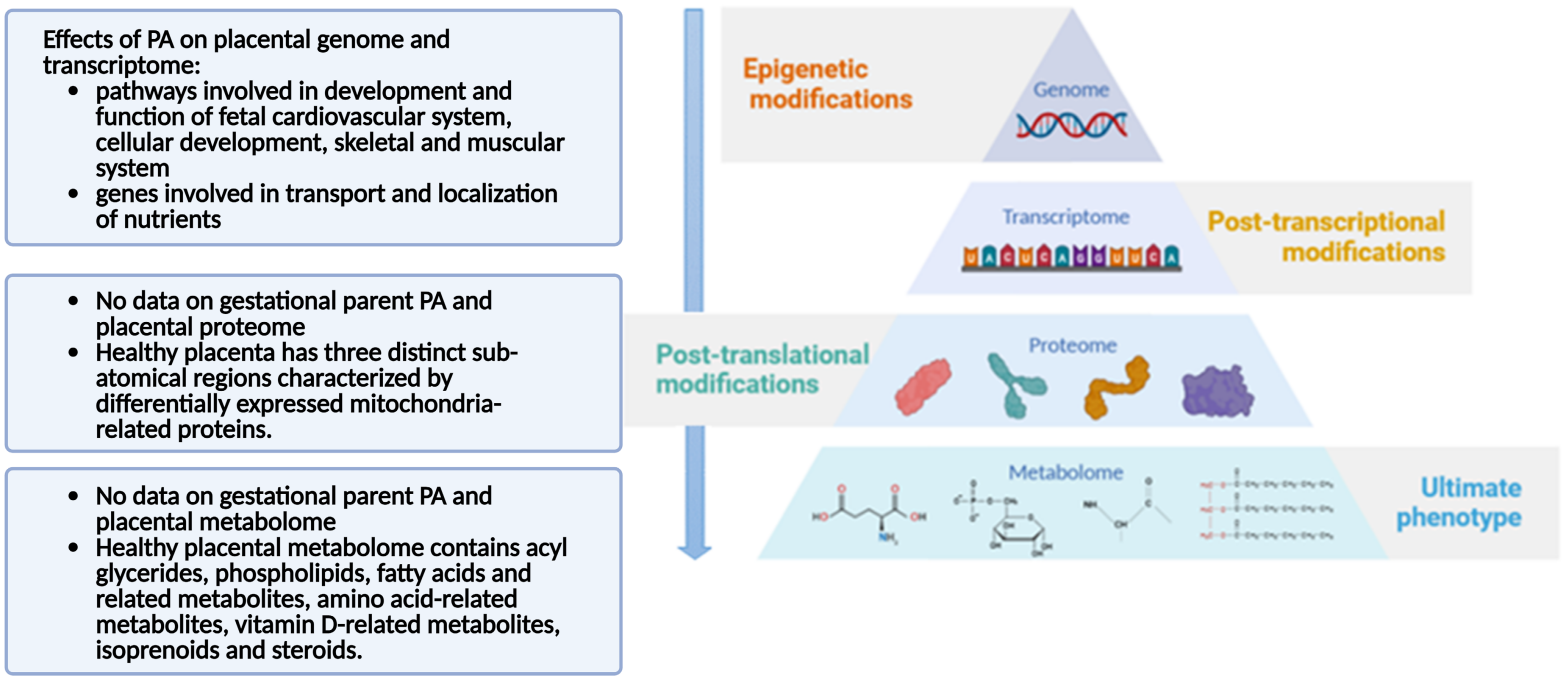
Summary of available ‐omics data on how gesP physical activity influences placental biology. gesP, gestational parent.

Placenta “‐omics” have gained increasing attention in the literature over the past 15 years. Reflective of the disease‐centric focus of much placenta research, there is a scarcity in ‐omics studies describing the effects of gesP PA on human placenta from uncomplicated pregnancies. This section aims to summarize the available literature identify gaps in knowledge (Figure 3).

### 4.1 Transcriptomics and epigenomics

Recent transcriptomic evidence suggests that gesP PA significantly alters placental gene expression. Loiselle et al. reported significant expression changes in placental genes involved in the transport and localization of nutrients between active and non‐active gesP. Most notably, aquaporin genes (AQP1 and AQP9) had higher expression in the placentas of active individuals, suggesting an adaptive response of water and glycerol (Loiselle et al., 2020). Epigenetic modifications, including DNA methylation, play a significant role in regulating gene expression and function. In an ethnically and racially diverse cohort of gesP, PA before and during pregnancy was associated with several DNA methylation sites in the placenta (Zhao, Yeung, et al., 2022). Pathway analysis demonstrated the influence of PA on placental DNA methylation is linked to pathways associated with the development and function of the fetal cardiovascular system, cellular development, as well as skeletal and muscular system development and function (Nayak et al., 2016). The most significant pathway linking PA and placental epigenetic modification was identified as the cardiac hypertrophy signalling pathway, important in the periconception and early‐pregnancy periods (Zhao, Yeung, et al., 2022).

### 4.2 Metabolomics

Most metabolomic studies have predominantly utilized biofluids, including urine, serum, and plasma, as a minimally invasive technique. However, over the past 15 years, the use of organ‐specific metabolomics has gained momentum. The placenta offers a distinctive opportunity for investigating organ‐specific metabolomics, as term placenta can be readily examined post‐birth. A comprehensive review of placental metabolomic signatures was recently conducted by Mohammad, Bhattacharjee, et al. (2021). In brief, healthy human term placenta from uncomplicated pregnancies are characterized by metabolomes containing acyl glycerides, phospholipids, fatty acids, and related metabolites, amino acid‐related metabolites, vitamin D‐related metabolites, isoprenoids, and steroids (Dunn et al., 2012). It should also be noted that normal metabolomic variations exist within the anatomical structure of the placenta, where the gesP and fetal placental surfaces have significantly different metabolic signatures (Walejko et al., 2018). Of sizable importance is the fact that no research to date has explored the impact of gesP behaviors such as PA on placental metabolic signatures. At this time, only inferences can be made from studies where altered oxygenation of healthy placental tissue resulted in significant increases in threonic/erythritol acid, 2‐deoxyribos, and hexadecenoic acid (Dunn et al., 2009; Heazell et al., 2008). It is presumed that PA creates a momentary decrease in oxygen delivery to placenta tissue as the blood is shunted to muscle tissue; thus, changes in the levels of the same metabolites may be observed in response to decreased oxygen levels in the placenta.

### 4.3 Proteomics

The placental proteome is well studied. These reports are broadly classified in either the spatial proteomic profiling of placental development (Fisher et al., 2019; Heywood et al., 2017; Khorami Sarvestani et al., 2021; Tong et al., 2016; Vandré et al., 2012) or comparative proteome analysis of term placenta in uncomplicated versus diseased condition (e.g., PE) (Baig et al., 2014; Huuskonen et al., 2016; Yang et al., 2015) categories. Neither category thus far has reported on how gesP PA influences the proteome of the placenta. Current research characterizes the healthy term placenta as having three distinct sub‐anatomical proteomic profiles (gesP‐facing side, middle placenta, and fetal‐facing side), showcasing the incredible heterogeneity of the organ and its multimodal function. It is possible that the different protein profiles indicate that these regions may have unique roles; however, currently there is very little evidence to draw any further conclusions. (Heywood et al., 2017; Manna et al., 2023; Vandré et al., 2012). Mitochondria‐related differentially expressed proteins were predominantly downregulated on the parent‐facing side and upregulated on the fetal‐facing side of the placenta when compared to the middle, suggesting a gradient in energy production and utilization across the placenta (Manna et al., 2023). Furthermore, proteins associated with cell death and apoptotic processes were primarily downregulated in the parental and fetal sides when compared to the middle placenta region (Manna et al., 2023). A significant gap in the literature exists on the topic of gesP PA and its effects on the placental proteomic profile, especially regarding mitochondrial function and energy utilization.

## 5 LIPIDS AND MITOCHONDRIA

**FIGURE 4:**
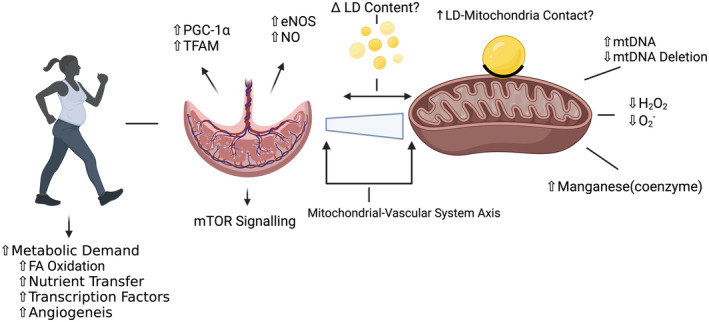
Visual representation of the current findings and proposed mechanisms of the effects of exercise on placental lipid metabolism and mitochondrial function. eNOS, endothelial nitric oxide synthase; FA, fatty acid; H_2_O_2_, hydrogen peroxide; LD, lipid droplets; mtDNA, mitochondrial deoxyribonucleic acid; mTOR, mammalian target of rapamycin; NO, nitric oxide; O_2_^−^, superoxide; PGC‐1⍺, peroxisome proliferator‐activated receptor‐gamma coactivator‐1alpha; TFAM, mitochondrial transcription factor A.

A large body of evidence suggests that PA can improve metabolic profiles, lipid metabolism, and mitochondrial function in tissues such as skeletal muscle (Hood, 2009). It has been established that several aspects of mitochondrial function are altered in response to PA, including mitochondrial biogenesis (Hood, 2009; Perry & Hawley, 2018), oxidative and antioxidant capacity (Carlsohn et al., 2008; Huertas et al., 2017; Lira Ferrari & Bucalen Ferrari, 2011; Powers et al., 2014), fatty acid oxidation (Fritzen et al., 2020; Jong‐Yeon et al., 2002), and mitochondrial DNA (mtDNA) transcription (Eluamai & Brooks, 2013). Placenta mitochondria undergo functional and structural changes throughout pregnancy, (Holland et al., 2017) and dysregulations have been linked to pathologies such as preeclampsia, metabolic disease, and FGR (Hebert & Myatt, 2021; Smith et al., 2021). Thus, a mechanism of PA‐derived pregnancy benefits may be through regulating mitochondria structure and function (Figure 4) (Bhattacharjee, Mohammad, & Adamo, 2021; Davenport, Meah, et al., 2018; Davenport, Ruchat, et al., 2018; Ferraro et al., 2012).

The limited number of studies investigating PA‐derived effects on placental mitochondria have indicated potential benefits. One study showed that PA during pregnancy resulted in greater mtDNA copy number with fewer deletions, and increased levels of manganese, which mitigates oxidative stress (Aparicio et al., 2023). Another study highlighted increased endothelial nitric oxide synthase (eNOS) and NO production along with reduced oxidative stress markers in active gesP (Ramírez‐Vélez et al., 2013). These findings suggest benefits for vascular function, mitochondria bioenergetics, and antioxidant capacity in the placenta. Due to the role of mitochondria in angiogenesis, the mitochondria–vascular axis is essential in placenta development (Reichard & Asosingh, 2019). Despite established relationships in other tissues, the effects of PA on mitochondrial dynamics in the placenta remains relatively unknown. Understanding this aspect is crucial for addressing diseases linked to mitochondrial dysfunction (Tanaka et al., 2020).

Mitochondrial genesis in the placenta is thought to influence nutrient transport and metabolism during pregnancy. In animal models, key mitochondrial biogenesis markers, such as PGC‐1α and mitochondrial transcription factor A, have been shown to change with gesP PA (Laker et al., 2021; Mohammadkhani et al., 2023). Work from our lab has shown that PA is associated with differences in placental mTOR molecules (Brett et al., 2015). As mTOR is a key regulator of mitochondrial function (Morita et al., 2015), alterations may contribute to the observed changes in mitochondrial functions surmised above.

The role of lipids in ATP production within placental mitochondria remains unclear, as does the impact of PA on placental lipid availability. However, preliminary evidence suggests that PA may alter lipid availability in cord blood. A 2020 study found higher levels of cord blood HDL‐c observed in active gesP compared to inactive controls, which may reflect alterations in placental lipids due to the shared vasculature (Collings et al., 2020).

One potential mediator of this process is the storage and release of lipids from lipid droplets (LD). A recent study from our lab found no total placental LD quantity differences between active and inactive participants with uncomplicated pregnancies (Cooper et al., 2023). Yet, physically active participants with insufficient gestational weight gain showed a greater LD coverage on the fetal placental side, suggesting that PA promotes the accumulation of LD at the fetal side in cases of inadequate weight gain. While PA boosts LD–mitochondria interactions in skeletal muscle (de Almeida et al., 2023), with increased contact between LD and mitochondria facilitating increased direct transfer of fatty acids (FA) to the mitochondria for oxidation, this interaction in placental tissue has yet to be explored. Further, proteins coating LD such as the perilipin (PLIN) family are integral in FA storage and release, and findings in skeletal muscle suggest differential expression in response to PA (Peters et al., 2012; Pourteymour et al., 2015). Additionally, PLIN members, particularly PLIN2, are expressed in the placenta (Bildirici et al., 2018). Accumulation of LD and altered PLIN levels have been found in the placenta of individuals with obesity or those who have experienced obesity and trophoblastic hypoxia/injury (Bildirici et al., 2018; Hirschmugl et al., 2017). Like obesity and hypoxia, PA is a potent metabolic and vascular stimulus, making it likely that gestational PA modifies LD function in tandem with PLIN family members. Thus, future study of the presence of this phenomenon within the placenta is warranted. Given the promising research on mitochondrial adaptations in other tissues, the relationship between gestational PA, placental mitochondria, and lipid metabolism is an area open for investigation.

## 6 IMMUNE SYSTEM CHARACTERISTICS

**FIGURE 5:**
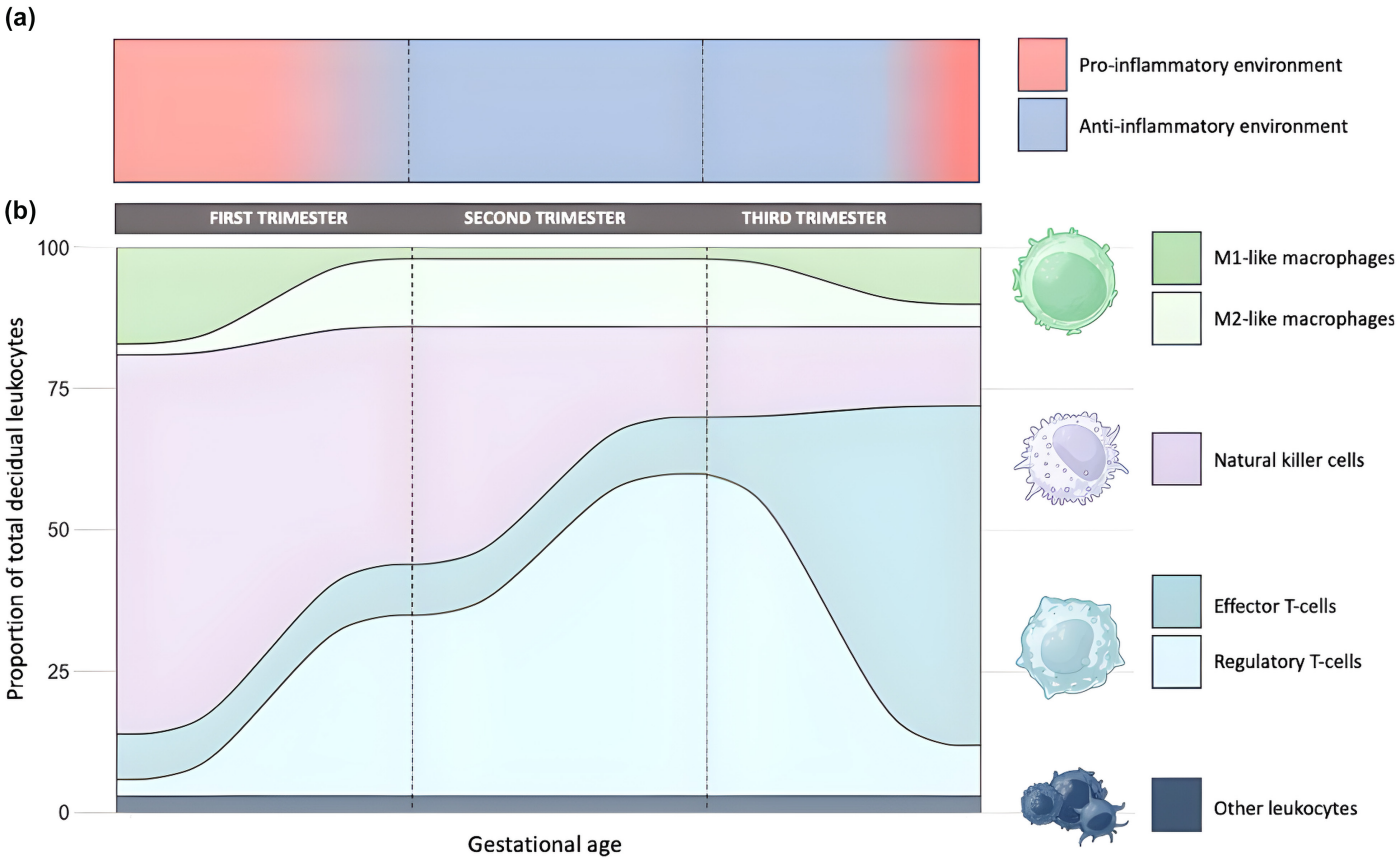
Temporal regulation of the placental immune system. a) predominant inflammatory profile of the placenta. b) relative proportions of leukocytes throughout gestation.

Leukocytes are vital for placenta development and homeostasis, representing 40% and 15% of cells in the decidua and chorion, respectively (Bulmer et al., 1991; Ingman et al., 2010). It has been previously reported that there are trimester‐specific inflammatory signatures (Dekel et al., 2010; Geldenhuys et al., 2018; Goudreau et al., 2021; Mor et al., 2011, 2017; Mor & Cardenas, 2010); as such, it is important to recognize that neither pro‐ nor anti‐inflammatory effects are inherently beneficial or detrimental. Rather, it is dysregulations of the tightly controlled system that contribute to adverse outcomes (Figure 5).

Within the chorion, Hofbauer cells (HBCs) are the only leukocytes of fetal origin and play important roles in pregnancy maintenance and progression (Fakonti et al., 2022; Thomas et al., 2021; Zulu et al., 2019). Like other tissue‐resident macrophages (TRMs), HBCs have diverse polarization states. Uncomplicated pregnancies lack classic pro‐inflammatory M1 HBCs; the majority are M2a and M2c (CD206+), with smaller amounts of M2b (CD206−) (Schliefsteiner et al., 2017). Although typically considered anti‐inflammatory, M2b macrophages share pro‐inflammatory traits with M1 populations and are linked to conditions like GDM. (Wang et al., 2019). Despite established effects on TRM polarization and function, the connection between improved outcomes in gestational PA and HBCs has been largely overlooked. Prior work by our lab showed active gesP have larger proportions of CD206+ HBCs, but lower CD206 protein expression (Goudreau, Everest, et al., 2023). As mRNA levels were not significantly different, it is possible that PA may posttranslationally downregulate CD206 in HBCs, increasing regulatory properties and mitigating adverse effects (e.g., fibrosis) (Goudreau, Everest, et al., 2023).

Unlike the chorion, there are diverse decidual leukocyte populations. In early‐pregnancy, decidual leukocytes are predominantly effectors of innate immunity. Natural killer cells (dNK) and macrophages (dMɸ) dominate the population, facilitating the pro‐inflammatory environment necessary for implantation (Faas & De Vos, 2017). Reduced dNK or overrepresentations of M2‐like dMɸ can lead to implantation failure and repeated miscarriages (Babayeva et al., 2020; Zhang et al., 2022). Conversely, under‐expression can lead to systemic inflammation in gesP, causing common first trimester symptoms (e.g., nausea and fatigue) or more serious complications (Kalagiri et al., 2016; Mor & Cardenas, 2010; Zhao et al., 2022). As PA reduces systemic inflammation across various conditions, gestational PA may help alleviate early‐pregnancy symptoms. Consistent with this notion, a surplus of lay accounts link PA with reduced nausea, improved sleep, and increased energy levels. While peer‐reviewed publications are limited, preliminary studies report similar results (Connolly et al., 2019; Owe et al., 2009, 2019). Likewise, PA is associated with decreased incidences of preeclampsia, hypertensive disorders, GDM, and hyperemesis gravidarum (Davenport, Ruchat, et al., 2018; Owe et al., 2019; Zhu et al., 2022). Following successful implantation, an anti‐inflammatory environment is established by the augmented population of M2‐like dMɸ, and the decreased numbers and cytotoxicity of dNK. This shift is crucial, as sustained inflammation threatens fetal development (Co et al., 2013). As regular PA favors M2‐like phenotypes in TRMs, including HBCs, it may support the shift to an anti‐inflammatory environment. Adaptive leukocytes in the decidua, including T‐cells (dTC), also shape trimester‐specific inflammatory profiles. The few dTC in early pregnancy are pro‐inflammatory effector cells (Lissauer et al., 2017). Effector dTC persist in midpregnancy to combat potential pathogens; however, the growing population predominantly contains regulatory T cells (dTreg) that support fetal development and mitigate the risk of miscarriage or preterm birth (Salvany‐Celades et al., 2019). Regular PA has been shown to promote dTreg mobilization, proliferation, and differentiation (Dorneles et al., 2020; Yeh et al., 2014), potentially regulating midpregnancy placental inflammation. Further studies should explore the relationships between PA, placenta leukocytes, and pregnancy outcomes.

## 7 ANGIOGENESIS

Vasculogenesis and angiogenesis continuously build the placenta vasculature required for nutrient and waste exchange at the parental‐fetal interface. Previous research shows that regular gestational PA stimulates midpregnancy placenta growth, and increases absolute and relative vascular volume at term (Bergmann et al., 2004). Molecular mediators of angiogenic pathways have been examined in the relationship with PA. A 2021 study conducted by Hardy et al. found that the term placenta of participants who were enrolled in an exercise routine between 16 and 20 weeks' gestation had increased expression of ANG1 at both mRNA and protein levels (Hardy et al., 2021). Likewise, work by our lab found that both the mRNA and protein expression of VEGF and its receptor VEGFR‐1 were increased in participants active during midpregnancy (Bhattacharjee, Mohammad, Goudreau, & Adamo, 2021). Unexpectedly, a subsequent study found that low molecular weight (LMW)‐FGF2, the secreted angiogenic isoform, was lower in active individuals (Goudreau, Tanara, et al., 2023). As, compared to FGF2, VEGF‐driven angiogenesis is linked to reduced inflammation and increased vascular, we suggest that the interplay of elevated VEGF and reduced LMW‐FGF2 may enhance capillary permeability, optimizing gas and nutrient exchange, while preserving the necessary anti‐inflammatory environment (Cao et al., 2004). Knowing VEGF and FGF2 both act in RTK pathways, we investigated the RTK‐regulatory factor SPRY2 (Hanafusa et al., 2002). Interestingly, SPRY2 displayed no discernible differences, suggesting that in uncomplicated pregnancies, molecular safeguards against over‐ or under‐active pathways are highly conserved regardless of PA. It appears that there is an “optimal” window, with studies reporting that morphological and molecular advantages are associated midpregnancy PA. Both HBCs and dMɸ have been implicated in this relationship due to their expression of angiogenic factors, proximity to developing vasculature, and heightened abundance during mid‐gestation (Zhao et al., 2022).

## 8 NEURODEVELOPMENT AND MENTAL HEALTH

While the existing literature provides robust evidence supporting the benefits of PA during pregnancy on neurodevelopment (ND), PA during pregnancy was historically viewed as harmful. A seminal 1996 study found that, contrary to their hypothesis, the children of active gesP performed better on tests of general intelligence and oral language skills at 5 years of age (Clapp, 1996). Human studies have continuously demonstrated associations between parental PA and offspring ND benefits (Domingues et al., 2014; Jukic et al., 2013; Nakahara et al., 2021; Niño Cruz et al., 2018; Silvente Troncoso et al., 2022), consistent with animal models (Akhavan et al., 2008; Bustamante et al., 2013, 2020; Klein et al., 2018; M Akhavan et al., 2013; Parnpiansil et al., 2003; Robinson & Bucci, 2014). Gestational PA confers advantageous changes in placenta morphology, including increased placenta functional volume, higher ratios of parenchymal to non‐parenchymal tissues, and improved angiogenesis (Baena‐García et al., 2023; Clapp et al., 2002; Kubler et al., 2022). These PA associations are linked to increased intelligence and reduced psychiatric risks in offspring. As previously discussed (section 6.0), PA may benefit angiogenesis, reducing risks for conditions that adversely affect ND, including placenta insufficiency (PI) and FGR (Arroyo & Winn, 2008; Cetin & Taricco, 2010). PI is associated with developmental delays; lower scores for cognitive, motor, and language performance; and the development of autism spectrum disorders (Burger et al., 2023; Lahti‐Pulkkinen et al., 2018; Misra et al., 2012; Segar et al., 2023; Walker et al., 2015). FGR similarly impairs ND, resulting in cerebral palsy and epilepsy; learning and attention disabilities; neurobehavioral difficulties; and frontal lobe dysfunction (Geva et al., 2006; Wixey et al., 2017).

**FIGURE 6:**
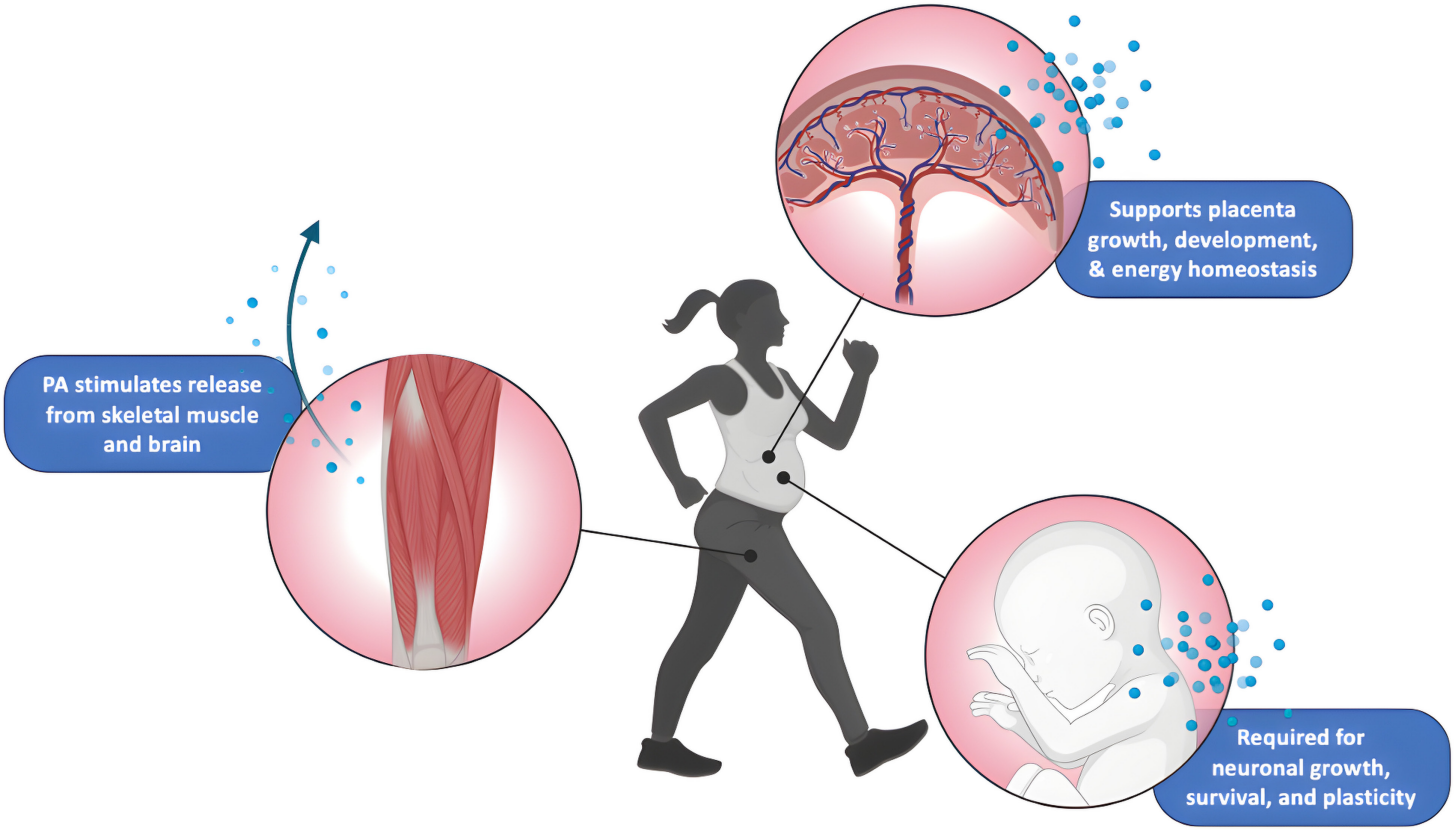
Established interactions between PA and BDNF in pregnancy. PA, physical activity.

Brain‐derived neurotrophic factor (BDNF) is a neurotransmitter vital for neuronal growth, survival, and plasticity (Bathina & Das, 2015). It also has pregnancy‐specific effects, contributing to placenta development, fetal growth, and energy homeostasis (Marchese et al., 2021; Prince et al., 2017). One study reported that first and second trimester PA increase parental BDNF (Rojas Vega et al., 2011), though its ability to cross the placenta is debated (Dingsdale et al., 2021; Granitzer et al., 2023; Sonmez et al., 2019). Neonates and placentas with significantly decreased BDNF levels have been identified in conditions that are mitigated by PA, including GDM; prepregnancy and gestational obesity; emergent caesarean sections; and late‐ or post‐term delivery (Prince et al., 2017; Ruchat et al., 2018). Pregnant parents with medicated psychiatric conditions exhibit lower umbilical cord serum BDNF levels, possibly contributing to the intergenerational cycle of mental illness (Flöck et al., 2016). It remains unclear whether this relationship is influenced by the disorders themselves or the medications used for treatment. While initial findings suggest a potential role in mitigating mental health risks (Figure 6), further research is needed to establish causation and underlying mechanisms, including placental changes, angiogenesis, and BDNF modulation.

## 9 CONCLUSIONS AND FUTURE DIRECTIONS

The work by our lab and others has uncovered unique features of placenta biology associated with gesP who are physically active during pregnancy. These presumably advantageous differences relate to nutrient transport, immune and metabolic function, placenta morphology/vascularization, various ‐omics characteristics, and fetal neurodevelopment. Despite the volume of work reviewed here, there are still knowledge gaps regarding the impact of gesP PA on key placental functionality and behaviors. Future research should continue to explore the influence of PA throughout pregnancy on the placental ‐omic profiles, and their cross talk, as those are the functional end phenotypes of the placenta and are translated directly to the fetus. There is a paucity of literature examining the contributions of PA on the placental proteomic profile, particularly in terms of mitochondrial function and energy utilization. There is a need for studies to investigate the links between gestational PA, placental mitochondria, and lipid metabolism, considering the already existing and promising research on PA‐related mitochondrial adaptations in other tissues. Although initial findings have demonstrated a role for PA during pregnancy in mitigating mental health risks in both parent and offspring, research to date has not established the causation nor underlying mechanisms, including placental changes, angiogenesis, and modulation of BDNF. The placenta, a finely tuned organ of critical importance, can adapt to and reflect the environments to which gesP and fetus are exposed; research is only beginning to unravel the role of PA in shifting the placental environment to one geared toward success.

## FUNDING INFORMATION

Much of the work discussed in this review is related to funding from CIHR (MOP‐ 142298) and NSERC (RGPIN‐2017‐05457) awarded to KBA. AC and MM both received a Canadian Graduate Student Award from CIHR and an Ontario Graduate Student Award (OGS).

## CONFLICT OF INTEREST STATEMENT

The authors have no conflict of interest to declare.
